# Relationship between dietary live microbe intake and the prevalence of COPD in adults: a cross-sectional study of NHANES 2013–2018

**DOI:** 10.1186/s12890-024-03045-2

**Published:** 2024-05-09

**Authors:** Dongbo Zhou, Baimei He, Qiong Huang, Siqi Li, Wenbin Nan, Qiong Chen, Qiao Yu

**Affiliations:** 1 Department of Geriatric Respiratory and Critical Care Medicine, Xiangya Hospital, Central South University, Changsha, 410008 China; 2 Department of Geriatric Medicine, Xiangya Hospital, Central South University, Changsha, 410008 China; 3 National Clinical Research Center for Geriatric Disorders, Xiangya Hospital, Central South University, Changsha, 410008 China; 4 Department of Emergency Medicine, Second Xiangya Hospital, Central South University, Changsha, 410011 China

**Keywords:** COPD, Prevalence, Live microbe, NHANES

## Abstract

**Objective:**

To explore the potential association between dietary live microbes and the prevalence of Chronic Obstructive Pulmonary Diseases (COPD).

**Methods:**

In this cross-sectional study, data of 9791 participants aged 20 years or older in this study were collected from the National Health and Nutrition Examination Survey (NHANES) between 2013 and 2018. Participants in this study were classified into three groups according to the Sanders’ dietary live microbe classification system: low, medium, and high dietary live microbe groups. COPD was defined by a combination of self-reported physician diagnoses and standardized medical status questionnaires. Logistic regression and subgroup analysis were used to assess whether dietary live microbes were associated with the risk of COPD.

**Results:**

Through full adjustment for confounders, participants in the high dietary live microbe group had a low prevalence of COPD in contrast to those in low dietary live microbe group (OR: 0.614, 95% CI: 0.474–0.795, and *p* < 0.001), but no significant association with COPD was detected in the medium and the low dietary live microbe groups. This inverse relationship between dietary live microbe intake and COPD prevalence was more inclined to occur in smokers, females, participants aged from 40 to 59 years old and non-obese participants.

**Conclusion:**

A high dietary live microbe intake was associated with a low prevalence of COPD, and this negative correlation was detected especially in smokers, females, participants aged from 40 to 59 years old and non-obese participants.

## Introduction

Chronic obstructive pulmonary disease (COPD) is a heterogeneous lung condition characterized by chronic respiratory symptoms, chronic inflammation and oxidative stress are the main pathogenesis of COPD, meanwhile autoimmune abnormality and airway dysbiosis also play an important role in its development [1, 2]. COPD is now one of the top three causes of death worldwide, more than 3 million people died of COPD in 2012 accounting for 6% of all deaths globally [3, 4]. COPD represents an important public health challenge that is both preventable and treatable [1]. Early detection and preventive interventions are crucial to control and delay progression of COPD [5]. COPD is caused by exposure to tobacco smoking and the inhalation of toxic particles and gas, host factors and genetic mutation, studies in recent years have found that diet is also involved in the development of COPD [6–9].

Our healthy existence is intricately reliant on the gut microbiota, or microbiome, which established a close symbiotic relationship with our body [10, 11]. Dysbiosis of gut microbiota is considered as an important component in the pathophysiology of COPD, and gut microbiota was involved in the exacerbation of COPD [12, 13]. Dietary supplementation of probiotics can prevent airway inflammation and lung damage in COPD mice, suggesting its potential therapeutic value for COPD [9, 14]. A wealth of studies have shown foods containing live microbes improve gut function and reduce risks of chronic disease [10, 15–17]. It’s reported that high consumption of fruits and vegetables, which provides high amount of live microbes, is associated with reduced COPD incidence [18–20]. It is not clear whether dietary live microbes yielded positive outcomes in COPD. The purpose of our study was to investigate the association between dietary live microbes and COPD prevalence based on surveillance data from the National Health and Nutrition Examination Survey (NHANES).

## Materials and methods

### Data source and study population

NHANES is a cross-sectional survey used to assess the health and nutrition of the US population. The study protocol was approved by the National Center for Health Statistics (NCHS) Research Ethics Review Board. The consent form was signed by every participant in the survey. The NHANES database is accessible without ethical or administrative approval.

**Fig. 1:**
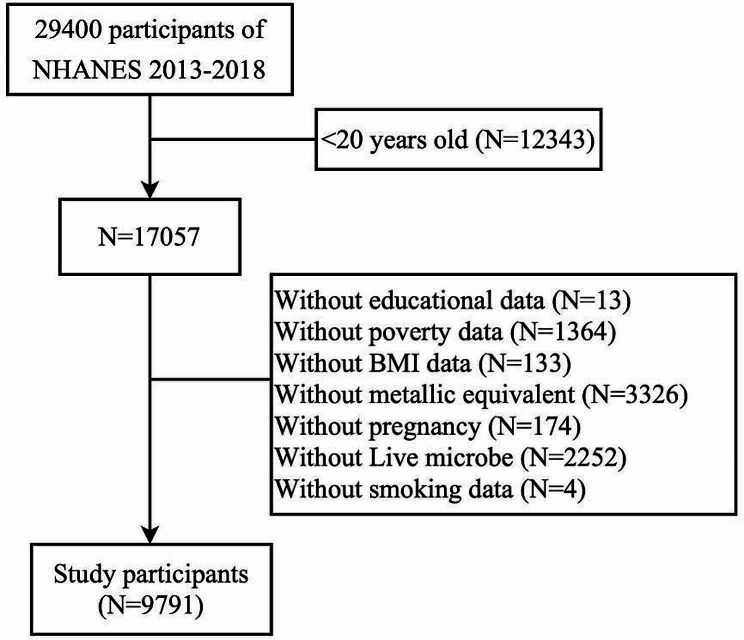
Flow chart of participants screening process

We enrolled participants from three cycles (2013–2014, 2015–2016, 2017–2018) of the NHANES. Patients were excluded as follows: (1) patients under the age of 20; (2) patients without dietary live microbe intake/COPD questionnaires data; (3) pregnant woman; (4) patients with missing data of educational level/body mass index/family poverty income ratio/metabolic equivalent /smoking status. A total of 29,400 participants were recruited in the present study, and 9791 participants were included in the further analyses after selecting participants with exclusion criteria. The selection procedure was summarized in Fig. 1.

### Live microbe intake

The dietary live microbe intake was estimated by using 24-h dietary recall data from NHANES. The food codes in the NHANES database were linked to the United States Department of Agriculture (USDA) to obtain the food composition and energy content data [21]. According to Sanders’ research, a team of four experts, relying on values reported in the primary literature, estimated the levels of live microbes (CFU/g) for 9,388 food codes across 48 subgroups in the NHANES database, and categorized microbial levels as low (< 10^4^ CFU/g), medium (10^4_^10^7^ CFU/g), or high (> 10^7^ CFU/g) based on the quantity of live microorganisms per gram of food. In short, the low class is mainly pasteurized foods, the medium class is mainly fresh fruits and vegetables that have not been peeled and the high class is fermented foods and probiotic supplements that have not been pasteurized. For these assessments, four experts relied on consulting the literature, authoritative reviews, and known effects of food processing (for example, pasteurization) on microbial viability. Any uncertain or conflicting data was resolved by reconciling within and between the teams, and external consulting with Fred Breidt, USDA Agricultural Research Service Microbiologist [19, 21].

### Covariates

The following covariates data were collected, including age, gender, ethnicity (Mexican American, Other Hispanic, Non-Hispanic White, Non-Hispanic Black, Other Races), education level (middle school or lower, high school, college or more), energy intake, family poverty income ratio (PIR), smoking status, past-year alcohol drinking, body mass index (BMI), metabolic equivalent (MET), diabetes mellitus, hypertension, asthma and dietary live microbes. PIR was estimated as the ratio of family income to the poverty threshold, and participants were divided into low-income (PIR < 1.3), medium-income (1.3 ≤ PIR < 3.5), and high-income (PIR ≥ 3.5) groups. BMI was calculated as weight (kg) divided by height (meters squared, m^2^). Smoking status was categorized as never, former, or current smoker. Past-year alcohol drinking included nondrinkers, 1–3 drinks/day and ≥ 4 drinks/day. MET refers to the oxygen consumption required to maintain resting metabolism and was classified as low, medium and high.

### Outcome definition

COPD is characterized by persistent (often progressive) airflow obstruction due to abnormalities in the airways (bronchitis, bronchiolitis) and/or alveoli (emphysema) that cause. Emphysema and chronic bronchitis are closely related to COPD. The participants were defined as COPD patients according to following questionnaire items: “Has a doctor or other health professional ever told you that you have COPD/Chronic bronchitis/Emphysema?” If Participants answered “yes” to any of the above questions were included in the COPD group, and those who answered “no” were placed in the non-COPD group. The outcome was thus converted to a dichotomous variable.

### Statistical analysis

Sample weight in NHANES was performed to fit in with the complex multistage cluster design. The normally distributed continuous variables were expressed as mean ± standard deviation (SD) and were analyzed using the t-test, while non-normally distributed continuous variables were presented as median (25th, 75th) and were analyzed using the Kruskal Wallis H test. The categorical variables were manifested as absolute values (n) with percentages (%) and were analyzed using the chi-square test. Participants in this study were divided into three groups (low, medium and high) according to Sanders’ dietary live microbe classification system. Univariate and multivariate logistic regression analyses were further applied to evaluate the relationship between dietary live microbe intake and the prevalence of COPD while adjusting for possible confounding factors, and three models were constructed. Crude Model was not adjusted for any covariates. Model I was adjusted for age and gender. Model II was adjusted for all covariates. To control the confounding effects of covariates on the relationship between dietary live microbes and COPD, the subgroup analysis were additionally performed according to age, sex, BMI and smoking status. All statistical analyses were performed by the R software version 4.2.0 and the Stata version 15.0. Statistical significance was set at *P* < 0.05 (two-sided).

## Results

### Baseline characteristics of participants

The characteristics of participants between COPD and non-COPD group were described in Table 1, the prevalence of COPD was 9.39%, 6.80% and 6.95% in the low, medium, and high dietary live microbe groups, respectively. Participants with COPD were more likely to be older, female, non-Hispanic White, smokers, poorer, with college or more degree, have higher BMI, have less energy intake, have hypertension, without diabetes and asthma. Furthermore, participants with COPD were more likely to consume fewer dietary live microbes (*p* < 0.001).

**Table 1:** The clinical characteristics of the study population with and without COPD

Variable	COPD		
	No	Yes	*p* value
NO. of participants	9030	761	
Age	46 (32, 61)	59(44, 69)	< 0.0001
Gender, n (%)			< 0.001
Female	4292(47.63)	413(59.33)	
Male	4738(52.37)	348(40.67)	
Ethnicity, n (%)			< 0.0001
Mexican American	1273(8.27)	44(2.84)	
Non-Hispanic Black	1922(10.59)	131(7.96)	
Non-Hispanic White	3467(66.74)	458(78.28)	
Other Hispanic	889(5.70)	60(4.06)	
Other Race - Including Multi-Racial	1479(8.70)	68(6.87)	
PIR, n (%)			< 0.0001
PIR < 1.3	2565(18.81)	315(30.48)	
1.3 ≤ PIR < 3.5	3421(34.55)	292(38.90)	
PIR ≥ 3.5	3044(46.64)	154(30.63)	
Education, n (%)			< 0.0001
Middle school or lower	1515(10.10)	164(14.92)	
High school	2013(22.29)	215(32.17)	
College or more	5502(67.61)	382(52.91)	
BMI	28(24.2, 32.6)	29.7(25.5, 35.6)	< 0.0001
Energy intake (kcal)	2009(1482, 2673)	1908(1370, 2484)	0.002
Metabolic equivalent, n (%)			0.01
Low	3008(30.79)	293(38.00)	
Medium	2993(35.37)	235(32.16)	
High	3029(33.84)	233(29.83)	
Smoking status, n (%)			< 0.0001
Never	5340(59.33)	209(27.00)	
Former	2056(23.91)	264(34.62)	
Current	1634(16.77)	288(38.38)	
Diabetes mellitus, n (%)			< 0.001
No	7574(88.27)	556(74.12)	
Yes	1456(11.73)	205(25.88)	
Hypertension, n (%)			< 0.0001
No	5538(65.86)	311(46.06)	
Yes	3492(34.14)	450(53.94)	
Asthma, n (%)			< 0.0001
No	7871(86.85)	437(59.32)	
Yes	1159(13.15)	324(40.68)	
Past-year alcohol drinking, n (%)			0.01
Nondrinkers	2719(23.73)	261(30.40)	
1–3 drinks/day	4979(60.44)	383(54.61)	
≥ 4 drinks/day	1332(15.83)	117(14.99)	
Dietary Live Microbes, n (%)			< 0.001
Low	3183(31.47)	330(38.15)	
Median	3506(35.99)	256(37.64)	
High	2341(32.54)	175(24.22)	

Table 2 summarized the baseline characteristics of these patients based on dietary live microbe intake. Participants with higher live microbe intake had a lower prevalence of COPD. Significant differences were observed in terms of age, gender, ethnicity, education level, PIR, BMI, energy intake, metabolic equivalent, past-year alcohol drinking, smoking status, and hypertension among the three groups (*P* < 0.001), instead of diabetes and asthma(*P* > 0.05).

**Table 2:** The clinical characteristics of the study population according to the different dietary live microbes

Variable	Low dietary live microbe group	Medium dietary live microbe group	High dietary live microbe group	*p* value
NO. of participants	3513	3762	2516	
Age	45 (31, 61)	48 (35,63)	47 (33,61)	< 0.0001
Gender, n (%)				< 0.0001
Female	1503(41.73)	1881(50.84)	1321(52.63)	
Male	2010(58.27)	1881(49.17)	1195(47.37)	
Ethnicity, n (%)				< 0.0001
Mexican American	418( 7.74)	650(10.57)	249( 4.94)	
Non-Hispanic Black	989(15.48)	727(10.05)	337( 5.69)	
Non-Hispanic White	1266(61.89)	1347(63.97)	1312(77.41)	
Other Hispanic	326(6.10)	366(5.76)	257(4.86)	
Other Race - Including Multi-Racial	514(8.80)	672(9.65)	361(7.10)	
PIR, n (%)				< 0.0001
PIR < 1.3	1272(26.62)	1026(18.39)	582(14.20)	
1.3 ≤ PIR < 3.5	1366(37.16)	1455(34.96)	892(32.50)	
PIR ≥ 3.5	875(36.23)	1281(46.66)	1042(53.31)	
Education, n (%)				< 0.0001
Middle school or lower	721(14.36)	689(10.70)	269( 6.29)	
High school	981(30.64)	769(20.82)	478(17.91)	
College or more	1811(55.00)	2304(68.48)	1769(75.81)	
BMI	28.7(24.5, 33.9)	28(24.3, 32.4)	27.6(23.9,32.2)	< 0.0001
Energy intake (kcal)	1942(1383, 2593)	1992.5(1468.25, 2634.25)	2103(1593.75, 2781.25)	< 0.0001
Metabolic equivalent, n (%)				0.0001
Low	1177(31.14)	1285(32.26)	839(30.47)	
Median	1045(30.78)	1310(37.55)	873(36.74)	
High	1291(38.09)	1167(30.19)	804(32.79)	
Past-year alcohol drinking, n (%)				< 0.0001
Nondrinkers	1144(27.16)	1185(25.30)	651(20.08)	
1–3 drinks/day	1746(52.52)	2073(60.39)	1543(67.07)	
≥ 4 drinks/day	623(20.31)	504(14.31)	322(12.86)	
Smoking status, n (%)				< 0.0001
Never	1815(51.24)	2239(58.88)	1495(60.40)	
Former	784(22.73)	937(26.54)	599(24.60)	
Current	914(26.04)	586(14.57)	422(15.00)	
Diabetes mellitus, n (%)				0.0589
No	2898(86.13)	3066(85.56)	2166(90.18)	
Yes	615(13.87)	696(14.44)	350( 9.82)	
Hypertension, n (%)				0.0094
No	2013(62.06)	2226(63.66)	1610(67.53)	
Yes	1500(37.94)	1536(36.34)	906(32.47)	
Asthma, n (%)				0.4773
No	2956(84.14)	3216(84.80)	2136(85.46)	
Yes	557(15.86)	546(15.20)	380(14.54)	
COPD, n (%)				0.0009
No	3183(91.12)	3506(92.24)	2341(94.36)	
Yes	330(8.88)	256(7.76)	175(5.65)	

### Association between dietary live microbe intake and COPD prevalence

Univariate logistic regression analysis revealed that participants in high dietary live microbe group (odds ratio [OR]: 0.637, 95% confidence interval [CI]: 0.445–0.912, and *p* = 0.016) had a low prevalence of COPD in contrast to those in the low dietary live microbe group in Table 3. There was no significant association between the medium dietary live microbe group and COPD prevalence, compared to the low dietary live microbe group.

**Table 3:** Univariate logistic regression analysis of COPD prevalence

	OR	CI	*P* value
Dietary Live Microbes			
Low	Reference		
Medium	0.891	0.690–1.149	0.356
High	0.637	0.445–0.912	0.016
Age (years)	1.04	1.029–1.051	< 0.001
BMI	1.018	1.005–1.032	0.009
Energy intake (kcal)	1.000	1.000–1.000	0.576
Gender			
Female	Reference		
Male	0.588	0.450–0.769	< 0.001
Ethnicity			
Mexican American	Reference		
Non-Hispanic Black	1.656	0.918–2.988	0.09
Non-Hispanic White	2.907	1.730–4.884	< 0.001
Other Hispanic	2.207	1.308–3.723	0.004
Other Race	1.950	1.033–3.682	0.04
PIR			
PIR < 1.3	Reference		
1.3 ≤ PIR < 3.5	0.726	0.515–1.025	0.067
PIR ≥ 3.5	0.456	0.300-0.695	< 0.001
Education			
Middle school or lower			
High school	0.995	0.686–1.441	0.976
College or more	0.722	0.471–1.109	0.13
Metabolic equivalent			
Low	Reference		
Median	0.920	0.680–1.244	0.572
High	0.930	0.692–1.250	0.614
Alcohol			
Nondrinkers	Reference		
1–3 drinks/day	0.825	0.614–1.109	0.191
≥ 4 drinks/day	1.083	0.670–1.751	0.735
Smoke			
Never	Reference		
Former	2.511	1.731–3.644	< 0.001
Current	5.189	3.599–7.482	< 0.001
Diabetes mellitus			
No	Reference		
Yes	1.373	0.965–1.953	0.076
Hypertension			
No	Reference		
Yes	1.078	0.816–1.424	0.583
Asthma, n (%)			
No	Reference		
Yes	5.115	3.770–6.940	< 0.001

Multivariate logistic regression analysis was used to illustrate the independent relationship between dietary live microbe intake and COPD prevalence in Table 4. In model I (adjusted for age and gender) the prevalence of COPD was significantly lower in high and medium dietary live microbe groups compared to low dietary live microbe group (*p* < 0.01). Through adjustment for model II (adjusted for model I plus ethnicity, education level, PIR, BMI, energy intake, MET, past-year alcohol drinking, smoking status, hypertension, DM and asthma), participants in high dietary live microbe group had a lower prevalence of COPD in contrast to low dietary live microbe group (OR: 0.614, 95% CI: 0.474–0.795, and *p* < 0.001), but no significant association with COPD was detected in medium and low dietary live microbe group.

**Table 4:** Association between dietary live microbe intake and COPD

Outcomes	Model	Low Dietary Live Microbes OR (95%)	Medium Dietary Live Microbes OR (95%)	High Dietary Live Microbes OR (95%)	*p*
COPD	Crude	1.00 (Reference)	0.891 (0.690–1.149)	0.637 (0.445–0.912)^*^	0.016
	Model 1	1.00 (Reference)	0.717 (0.570–0.902)^*^	0.517 (0.400-0.669)^*^	< 0.01
	Model 2	1.00 (Reference)	0.863 (0.698–1.067)	0.614 (0.474–0.795)^*^	< 0.001

### Subgroup analyses

To control the confounding effects of covariates on the relationship between dietary live microbe intake and the prevalence of COPD, subgroup analyses according to these covariates were conducted. As shown in Table 5, high live microbe level was significantly associated with a lower prevalence of COPD than low and medium live microbe level in crude model and adjusted models (model I and model II), and this association was only significant in smokers/former smokers. This negative association was not observed in never smokers. As presented in Table 6, a significant inverse correlation between live microbes and COPD prevalence were found in female participants in three models and male participants in crude model and model I. We found that live microbe level is inversely related to COPD prevalence in three models among participants aged from 40 to 59 years old (Table 7). This relationship was also present in the 20–39 age group and the 60–80 age group but only in crude model and the minimally adjusted model I. What’s more, a robustly negative relevance between live microbe level and the prevalence of COPD was confirmed in BMI < 30 kg/m^2^ group in Table 8. Conversely, this relevance was not significant in BMI ≥ 30 kg/m^2^ group.

**Table 5:** Association of dietary live microbe level with COPD in groups by smoking status

Live Microbe Group	Crude model	Model I	Model II
**Smokers /former smokers**			
Low	1.00	1.00	1.00
Medium	0.87(0.67,1.12)	0.72(0.54,0.95)	0.85(0.60,1.20)
High	0.67(0.49,0.92)	0.53(0.39,0.70)	0.63(0.42,0.95)
P for Trend	< 0.001	< 0.001	0.026
**Never smokers**			
Low	1.00	1.00	1.00
Medium	1.20(0.86,1.67)	0.99(0.70,1.40)	0.86(0.53,1.39)
High	0.74(0.45,1.21)	0.64(0.38,1.09)	0.64(0.35,1.18)
P for Trend	0.187	0.078	0.138

**Table 6:** Association of dietary live microbe level with COPD in groups by gender

Live Microbe Group	Crude model	Model I	Model II
**Female**			
Low	1.00	1.00	1.00
Medium	0.76(0.56,1.03)	0.68(0.49,0.94)	0.81(0.55,1.18)
High	0.56(0.40,0.79)	0.51(0.36,0.71)	0.56(0.34,0.93)
P for Trend	0.001	< 0.001	0.023
**Male**			
Low	1.00	1.00	1.00
Medium	0.92(0.67,1.26)	0.77(0.56,1.05)	0.98(0.65,1.49)
High	0.60(0.43,0.83)	0.53(0.38,0.74)	0.73(0.46,1.16)
P for Trend	0.003	< 0.001	0.181

**Table 7:** Association of dietary live microbe level with COPD in groups by age

Live Microbe Group	Crude model	Model I	Model II
**20–39 years**			
Low	Reference	Reference	Reference
Medium	0.76(0.48,1.19)	0.72(0.46,1.13)	0.93(0.55,1.56)
High	0.64(0.42,0.96)	0.61(0.41,0.92)	0.83(0.49,1.41)
P for Trend	0.029	0.016	0.470
**40–59 years**			
Low	Reference	Reference	Reference
Medium	0.65(0.38,1.10)	0.59(0.34,1.01)	0.70(0.40,1.26)
High	0.46(0.31,0.69)	0.42(0.28,0.62)	0.46(0.27,0.78)
P for Trend	< 0.001	< 0.001	0.005
**60–80 years**			
Low	Reference	Reference	Reference
Medium	0.89(0.68,1.18)	0.89(0.67,1.18)	1.10(0.67,1.83)
High	0.61(0.42,0.88)	0.60(0.42,0.87)	0.84(0.48,1.45)
P for Trend	0.007	0.005	0.491

**Table 8:** Association of dietary live microbe level with COPD in groups by BMI

Live Microbe Group	Crude model	Model I	Model II
**BMI (< 30 kg/m** ^**2**^ **)**			
Low	1.00	1.00	1.00
Medium	0.87(0.66,1.15)	0.71(0.53,0.95)	0.90(0.63,1.27)
High	0.51(0.33,0.78)	0.41(0.27,0.64)	0.58(0.34,0.98)
P for Trend	0.002	< 0.001	0.036
**BMI (≥ 30 kg/m** ^**2**^ **)**			
Low	1.00	1.00	1.00
Medium	0.88(0.62,1.25)	0.75(0.52,1.08)	0.85(0.56,1.29)
High	0.82(0.57,1.20)	0.73(0.51,1.05)	0.70(0.40,1.23)
P for Trend	0.293	0.085	0.193

## Discussion

Considering high prevalence and poor prognosis of COPD, early prevention can effectively minimize morbidity and reduce long-term social burden [5, 22]. In this cross-sectional study among American adults, we observed that dietary patterns with high live microbe intake contributed to a lower prevalence of COPD. What’s more, an independent inverse correlation between live microbes and prevalence of COPD was confirmed in participants with a history of smoking, female, aged from 40 to 59 and BMI < 30 kg/m^2^.

The human microbiota comprises trillions of microorganisms per individual, and changes in the composition and function of resident microbiota are associated with a number of diseases [23]. An altered or dysbiotic microbiota can contribute to inflammation in the whole body and pathogenic bacterial infection, including inflammatory bowel disease, COPD, and type 2 diabetes [13, 24–26]. The manipulation of gut bacteria with probiotics may be an attractive therapeutic strategy to strengthen the intestinal barrier and ameliorate the systemic inflammatory state in multiple diseases [25–27]. Several studies implied that probiotics are shown to reduce lung inflammation and improve airways remodeling in experimental COPD animal models and probiotics supplementation reduce circulating inflammatory cytokines in COPD patients [28, 29]. There is no doubt about the usefulness of probiotics. In addition to probiotic supplements, many foods also contain live microbes (for example, yogurt and kimchi), which can improve metabolic and immune health [15]. Foods with live microbes, including fresh vegetables, fruits, fresh fruit juices, beverages, condiments, sauces and fermented foods, were associated with a lower blood pressure, BMI, waist circumference, plasma glucose, C-reactive protein, insulin, and triglyceride levels, along with a higher HDL cholesterol level [10, 15, 16, 30]. Considering that the availability of food is higher than that of probiotic supplements, the research on live microbes in food has gradually come into the view of scientists in recent years. The consumption of live microbes has also been directly linked to reduce the incidence and duration of common upper respiratory infections and gastrointestinal infections [29–32]. Diet has emerged as a major driver of the composition and function of the live microbes [30–32]. Our study provides evidence that high dietary live microbe intake decreases the prevalence of COPD.

Possible mechanisms for the relationship between the high dietary live microbes and COPD are as follows. First, live safe microbes obtained from daily intake in the diet may “engage” with the mucosal surfaces of the digestive tract, fine-tuning the immune system, bolstering gut function, and reinforcing the ability of the human symbiont to mitigate susceptibility to the development of chronic diseases [10, 17, 33]. Second, it is known that the pathogenesis of COPD may involve oxidative stress and inflammation, fermented foods, high fruit and vegetable consumption reduced levels of inflammation parameters and increased levels of antioxidant defense [10, 18, 34–38]. Third, evidence from the gut-lung axis research suggested that COPD might be prevented or at least can be ameliorated by regulating gut microbial ecosystem through manipulation of gut microbiota, high dietary live microbes can lead to balance gut microbiota and enhance immune response [13, 39, 40].

What’s more we found that this inverse correlation between live microbe intake and the prevalence of COPD was detected especially in smokers, females, participants aged from 40 to 59 years old and non-obese participants. COPD is caused by interactions between environmental exposures, genetic susceptibility, and lifestyle and host factors [8, 41]. Cigarette smoking is the main risk factor of COPD [42, 43]. In the subgroup analysis of smokers and never smokers, we found an inverse correlation between live microbe intake and the prevalence of COPD in smokers including former smokers and current smokers. The abundance and growth of gut microbiome are reduced by cigarette smoke exposure in humans and rats, and cigarette smoking has a strong influence on the gut microbiome in COPD [42, 44]. Budden et al. found that probiotic Bifidobacterium alleviated cigarette smoke-induced inflammation in mice [45]. Moreover, probiotics may restore natural killer cell activity which is lowered in smokers [46]. These researches suggested gut microbiome’s potential therapeutic value for smoking related COPD and liver microbe can improve the adverse effects of smoking. In our study we demonstrated that higher levels of live microbe intake are beneficial in reducing COPD incidence in smokers.

The associations between live microbe intake and COPD prevalence were different in men and women. Whereas higher live microbe intake was independently associated with lower COPD prevalence in women, this significant association was not observed in men. Sex can influence the complexity and diversity of microbes that we harbour in our gut, and reciprocally that our gut microbes can directly and indirectly influence sex steroid hormones and central gene activation [47–49]. In abdominally female obese individuals, consumption of Bifidobacterium improves anthropometric adiposity biomarkers [50]. Childhood probiotic supplementation may selectively decrease body mass index-for-age z-score in female adolescents [51]. Currently only a handful of studies have looked at male vs. female differences upon probiotic administration, but these results implied that probiotic supplements or live bacteria intake may be more predisposed to affect females [52, 53]. Similarly, our results demonstrated that there may be some gender-specific variances in terms of live microbe mediated effects, and the specific mechanism needs further study.

Age is an important variable affecting gut microbiome in terms of gut microorganism population and diversity [52]. Ghosh et al. reported that the Lactobacillus rate in the elderly was significantly higher than the Child/Teen/Young/Middle-aged groups in North America and European individuals [54]. The age of the host can likewise influence probiotic study outcomes in humans [55]. Kwok et al. demonstrated that response in the small intestine to probiotics supplementation is likely age-specific [56]. Liu et al. suggested that probiotic, prebiotic, or yogurt supplements may contribute to the prevention of chronic kidney diseases and relieve its progression risk, especially in older population who were aged 55 years or older [57]. Our study discovered that the inverse relationship between live microbe level and COPD prevalence was significant in people aged from 40 to 59 years old, not in 20–39 age group and the 60–80 age group. Although these results of studies have been inconsistent, these provided important evidence that responses in host to probiotics/live microbes were age-specific. Therefore, further large-scale investigation is required to assess differences in mechanism of probiotics and live microbes among different age groups.

Gut microbiota dysbiosis has been recognized as having key importance in subjects with BMI higher than 30 kg/m^2^ [58]. Six months supplementation with probiotic resulted in significant BMI reduction in overweight/obese individuals, and greater reduction was apparent in patients receiving higher dosages [59]. A meta-analysis of randomized clinical trials with 957 subjects, with a mean BMI of 27.6 kg/m^2^, showed that probiotic administration significantly reduced BMI by 0.27 kg/m^2^ [58]. Studies showed that compared to those with a normal BMI, subjects with low BMI had a higher prevalence of COPD, but those with BMI ≥ 22.0 kg/m^2^ had a 42% reduction in risk of COPD death [60, 61]. Given the role of BMI in the development of COPD and the association of BMI and probiotics, we found an interesting result that the negative relevance between live microbe intake and COPD was only observed at non-obese group (BMI < 30 kg/m^2^), instead of obese group (BMI ≥ 30 kg/m^2^). These findings manifested that there may be a complicated correlation among BMI, COPD and live microbes, and the specific mechanism and the different cut-off of BMI in each study needed to be further explored.

So far, there are few study focus on the association between dietary live microbes and the prevalence of COPD, this study explore this potential association in a large representative US population. There are several strengths in present study. First, we examine the effect of whole foods including live microbes, not only fermented foods or probiotics, on COPD prevalence. Second, our study used a nationally representative database in the United States from the NHANES, and all data was subjected to rigorous quality control to ensure their validity. Third, we applied subgroup analyses and obtained valuable results that there was an inverse correlation between live microbe intake and prevalence of COPD in participants with a history of smoking, female, aged from 40 to 59 and BMI < 30 kg/m^2^. 

Nevertheless, our study is also subject to several limitations. First, the NHANES is a cross-sectional study, we only observed the association, and it is incapable of establishing temporal and causative relationships between the factors being evaluated. Second, 24-h dietary recall data may be inaccurate due to recall bias and dietary live microbes can be affected by transportation, storage, and cooking. Third, due to various dietary behavior in different regions, the conclusion in this study only applies to Americans, and cannot be extended to other population. In addition, the self-reported dietary intakes and diagnosis of COPD/Chronic bronchitis/Emphysema resulted in an information bias. Therefore, further researches are required to investigate the association between live microbe intake and prevalence of COPD.

## Conclusions

Our study indicated that a high dietary live microbe intake was negatively associated with a low prevalence of COPD, especially in smokers, females, patients aged 40–59 years old and non-obese participants.

## Data Availability

All data analyzed in our study were extracted from NHANES and could be found at https://www.cdc.gov/nchs/nhanes/.

## Abbreviations

COPD: Chronic obstructive pulmonary disease
NHANES: National Health and Nutrition Examination Survey
NCHS: National Center for Health Statistics
USDA: US Department of Agriculture
BMI: Body mass index
FIR: Family income-to-poverty ratio
